# Neurodevelopmental Disorders in Offspring Conceived via In Vitro Fertilization vs Intracytoplasmic Sperm Injection

**DOI:** 10.1001/jamanetworkopen.2022.48141

**Published:** 2022-12-22

**Authors:** Huiwen Lo, Shih-Feng Weng, Eing-Mei Tsai

**Affiliations:** 1 Department of Obstetrics and Gynecology, Kaohsiung Medical University Chung-Ho Memorial Hospital, Kaohsiung, Taiwan; 2 Graduate Institute of Medicine, College of Medicine, Kaohsiung Medical University, Kaohsiung, Taiwan; 3 Department of Healthcare Administration and Medical Informatics, Kaohsiung Medical University, Kaohsiung, Taiwan; 4 Center for Medical Informatics and Statistics, Office of Research and Development, Kaohsiung Medical University, Kaohsiung, Taiwan; 5 Center for Big Data Research, Kaohsiung Medical University, Kaohsiung, Taiwan

## Abstract

**Importance:**

Intracytoplasmic sperm injection (ICSI), the most common type of assisted reproductive technology (ART), might damage the sperm or embryo. The implications of male infertility and ICSI for the neurodevelopmental health of offspring remain unknown.

**Objective:**

To analyze the risks of neurodevelopmental disorders in offspring of couples with male or female infertility with or without ICSI use.

**Design, Setting, and Participants:**

This cohort study was conducted in Taiwan and used information collected from the national population registry data set, national birth data set, and national ART data set for all live singleton births from January 1, 2008, to December 31, 2016. The follow-up period started from the date of birth until the diagnosis of a disorder or December 31, 2018, whichever occurred first. Data were analyzed from July 1, 2021, to August 1, 2022.

**Exposures:**

Male or female infertility with or without ICSI.

**Main Outcomes and Measures:**

The outcome was the incidence of autism spectrum disorder (ASD), attention-deficit/hyperactivity disorder (ADHD), and developmental delay in offspring with ART conception. Taiwan’s national population registry data set was used to identify ASD, ADHD, and developmental delay diagnosed in outpatient clinic and hospitalization records.

**Results:**

The study included 1 575 971 singleton births (mean [SD] age, 5.87 [2.60] years; 819 389 boys [52.0%]), of whom 1 568 257 (99.5%) had natural conception, 2111 (0.1%) had ART conception with male infertility, and 5603 (0.4%) had ART conception with female infertility. The risks of ASD (adjusted hazard ratio, 2.49; 95% CI, 1.61-3.84; *P* < .001) and developmental delay (adjusted hazard ratio, 1.92; 95% CI, 1.54-2.39; *P* < .001) in offspring with ART conception and ICSI use were significantly higher than those in offspring with natural conception. The same results were found in offspring of couples with either male or female infertility and ICSI intervention.

**Conclusions and Relevance:**

Results of this study suggest that male infertility was not associated with an increased risk of neurodevelopmental disorders in offspring. In both male and female infertility groups, ICSI had unfavorable implications for the neurodevelopmental health of offspring in terms of increased risks of ASD and developmental delay.

## Key Points

**Question:**

Are male infertility and intracytoplasmic sperm injection (ICSI) associated with an increased risk of neurodevelopmental disorders in offspring?

**Findings:**

In this cohort study of 1 575 971 singleton births, ICSI was associated with an increased risk of autism spectrum disorder (ASD) and developmental delay in offspring whose parents experienced infertility. Offspring of couples with either male or female infertility who did not receive ICSI intervention had no increased risk of ASD and developmental delay.

**Meaning:**

Findings of this study suggest that ICSI had unfavorable implications for the neurodevelopmental health of offspring in couples with either male or female infertility.

## Teaser

This cohort study assesses the incidence of neurodevelopment disorders, including autism spectrum disorder, attention-deficit/hyperactivity disorder, and developmental delay, in offspring of individuals who received assisted reproductive technology.

## Introduction

More than 10 million offspring have been conceived via assisted reproductive technology (ART) since the first successful birth using ART in 1978.^1^ However, there are concerns regarding the neurodevelopmental health of offspring born via ART. One concern is the implications of male infertility for the neurological health of offspring. Several studies have reported that males transmit epigenetic inheritance, and the epigenetic mechanism may regulate embryonic gene expression and affect the neurological health of their offspring.^2,3,4,5^ However, the association between male infertility and neurodevelopmental health of offspring remains unclear.^6^

Another concern is the association of intracytoplasmic sperm injection (ICSI) with the neurodevelopmental health of offspring. In contrast to sperm competing with each other to fertilize oocytes in conventional in vitro fertilization (IVF), selected sperm is injected manually into the oocytes in ART with ICSI. Morphologically normal sperm is selected via swim-up or density-gradient centrifugation methods during ICSI. This procedure is more invasive than conventional IVF.^7^ Intracytoplasmic sperm injection has become the most popular type of ART since its introduction in 1992.^8^ The popularity of ICSI in ART has increased from 20% in 2011 to approximately 70% currently.^9^ Intracytoplasmic sperm injection is used not only in cases of male infertility but also in those of female infertility. It is believed to decrease the fertilization failure rate and increase the ART success rate.^10^ However, studies have claimed that ICSI alters the epigenetic regulation of embryos and affects the health of offspring.^11,12^ The reasons given were that the risk of genomic defects in motile sperm increases after performing isolation methods for ICSI.^7^ This defective sperm might then be injected into oocytes. Oocytes are in a stressful environment during the procedure, such as prolonging operation time outside the incubation as well as slight alteration in temperature, gas concentration, and pH.^8^ However, for couples with male or female infertility, the association of ICSI use with offspring health remains unclear.

The risks of autism spectrum disorder (ASD), developmental delay, and attention-deficit/hyperactivity disorder (ADHD) have been described in offspring who were conceived via ART or ART combined with ICSI.^13^ Increasing evidence suggests that epigenetics plays a prominent role in the development of these disorders,^14^ along with other variables, such as sex and preterm delivery.^15,16^ However, there is limited evidence regarding the association of male infertility and ICSI with the risk of neurodevelopmental disorders in offspring. This cohort study was designed to analyze the risk of neurodevelopmental disorders in offspring of couples with male or female infertility with or without ICSI use.

## Methods

This retrospective cohort study was conducted in Taiwan and approved by the institutional review board of Kaohsiung Medical University Chung-Ho Hospital, which waived the requirement for informed consent because the data were encrypted and deidentified. We followed the Strengthening the Reporting of Observational Studies in Epidemiology (STROBE) reporting guideline.

More than 99% of the citizens of Taiwan have participated in the National Health Insurance program since 1995, and the national population registry data set is linked to the national ART and birth certification data set. Medical information in the national population registry data set is recorded at the time of visit to outpatient public or private clinics. Information has been recorded since 1998 for all patients receiving ART and since 1994 for all newborns with 20 or more weeks of gestation or weighing at least 500 g at birth.

We used the national ART data set to identify the basic profiles of patients who received ART and delivered singletons after treatment from January 1, 2008, to December 31, 2016. Next, we collected the basic birth profiles of offspring with natural or ART conception from the birth certification data set. We used the national population registry data set for the follow-up period for assessment of neurodevelopmental disorders in offspring, which started from the date of birth until the diagnosis of a disorder or December 31, 2018 (whichever occurred first).

### Participants

First, we selected couples with infertility who underwent ART from the national ART data set. Couples with female or male infertility, defined as an abnormality in sperm finding via the conventional semen parameter, such as number of sperm, morphology, and motility, were included. Couples with donated oocytes or sperm, embryo manipulations other than ICSI (ie, hatching, defined as thinning the zona pellucida to facilitate implantation; preimplantation genetic testing for aneuploidy, defined as assessing the genome of an embryo), incomplete records, and multiple live births were excluded. Additional information included age, number of retrieved oocytes, number of fertilized oocytes, biochemical pregnancies (early pregnancy with β-hCG [human chorionic gonadotropin] level increase), clinical pregnancies (with ultrasonographic confirmation of fetal heart beat), miscarriages, and live births.

Second, we selected a cohort of all live singletons with either natural or ART conception from January 1, 2008, to December 31, 2016, from the birth certification data set. The basic information included maternal risk factors during pregnancy (ie, diabetes, gestational diabetes, hypertensive disorder, unhealthy lifestyle habits [such as alcohol drinking, smoking, and drug addiction]), maternal complications during labor (ie, prolonged premature rupture of membrane >12 hours, placental abruption, placenta previa, postpartum hemorrhage, and fetal distress), and offspring data (gestational age, delivery method, sex, and 1- and 5-minute Apgar scores).

### Exposure

We evaluated the pregnancy outcome in patients who received ART based on the etiology of infertility, status of embryos, and presence or absence of ICSI. Next, we assessed neurodevelopmental outcomes in offspring based on the mode of conception and the etiology of parental infertility: (1) natural conception (control group), (2) ART conception associated with female infertility, and (3) ART conception associated with male infertility. We also divided singletons into 3 groups to identify the risks of ICSI in neurodevelopmental disorders in offspring: natural conception (control group), ART conception with ICSI, and ART conception without ICSI. We further defined subgroups of offspring conceived with ART based on the presence or absence of ICSI and parental infertility factor: (1) offspring conceived via ART with ICSI associated with female infertility (female with ICSI), (2) offspring conceived via ART without ICSI associated with female infertility (female without ICSI), (3) offspring conceived via ART with ICSI associated with male infertility (male with ICSI), and (4) offspring conceived via ART conception without ICSI associated with male infertility (male without ICSI).

### Outcomes

The outcome was the incidence of ASD, ADHD, and developmental delay in offspring with ART conception. We analyzed the risks of ASD (*International Classification of Diseases, Ninth Revision* [*ICD-9*] code 299 or *International Statistical Classification of Diseases and Related Health Problems, Tenth Revision* [*ICD-10*] code F84), ADHD (*ICD-9* code 314 or *ICD-10* code F90), and developmental delay (*ICD-9* code 315 or *ICD-10* codes F80-82 and R48.0) from the national population registry data set. The disorders must have been followed up at clinics at least twice a year using the same *ICD-9* and/or *ICD-10* diagnosis codes, and the same *ICD* codes must have been used more than 6 months apart.

### Covariates

Several confounders, including maternal or paternal psychiatric disorders, maternal or paternal age, offspring sex, and gestation status (full-term or preterm), were noted in this study. We classified maternal or paternal psychiatric disorder (*ICD-9* code 290-315 or *ICD-10* code F00-99) as the presence or absence of the disorder before offspring birth using the national population registry data set. The same *ICD-9* and/or *ICD-10* codes must have been used in follow-ups at clinics for at least twice a year and must have been used more than 6 months apart.

### Statistical Analysis

First, couples who received ART were grouped based on infertility factors and the presence or absence of ICSI. The ART outcome included fertilization rate, biochemical pregnancy rate, clinical pregnancy rate, miscarriage rate, and live birth rate. Baseline characteristics were analyzed by χ^2^ test, and ART outcome was assessed by regression model.

Second, we used inverse probability of treatment weighting (IPTW) to balance the baseline characteristics in offspring among the exposure and comparison groups by calculating the propensity score via a multinominal logistic regression analysis. We calculated the propensity score for each offspring with the following variables: paternal or maternal psychiatric disorder (presence or absence), risk factors during pregnancy, and complications during labor. Next, we weighted each offspring by the inverse of the probability of their treatment allocation and created the pseudo data set.^17^ A weighted χ^2^ test and standardized mean difference (SMD) were used to evaluate the balance of baseline characteristics among the 3 groups.

Third, we used the incidence of disease and Cox proportional hazards regression model (model 1) to assess the implications of infertility etiology or ICSI for ASD, ADHD, and developmental delay in offspring. Follow-up time started from the birth of the offspring to the diagnosis of a disorder or December 31, 2018, whichever occurred first. We calculated the total number of person-years for each offspring and presented the incidence of ASD, ADHD, and developmental delay as the disease rate per 1000 person-years. The risk regression models, subdistribution hazard ratios (HRs), 95% CIs, and *P* values were used to evaluate the incidence and likelihood of disease. We also created model 2 to adjust for the following covariates by regression model: maternal or paternal age at birth, gestation status (preterm or full-term), and offspring sex.

Fourth, we used the same methods to estimate the risk of ICSI in male and female infertility groups. Offspring in the natural conception group were used as the reference.

All statistical tests were performed using SAS, version 9.4 (SAS Institute). Statistical significance was set at a 2-sided *P* < .05. An SMD less than 0.1 defined the balance between the 2 groups. All HRs are presented as well. Data were analyzed from July 1, 2021, to August 1, 2022.

## Results

### Characteristics of the Study Population

We identified patients undergoing ART with male infertility or female infertility after excluding embryo manipulation with hatching and preimplantation genetic testing for aneuploidy, incomplete record of oocytes and embryos, and no embryo transfer. Next, we divided patients undergoing ART into subgroups based on fresh or frozen embryo transfer (Figure). In these subgroups, we evaluated the ART outcome and set patients without ICSI use as the control group. The results revealed that the rate of fertilization, biochemical pregnancies, clinical pregnancies, and live births did not improve in couples with female or male infertility with ICSI use (eTables 1 and 2 in Supplement 1).

**Figure.**
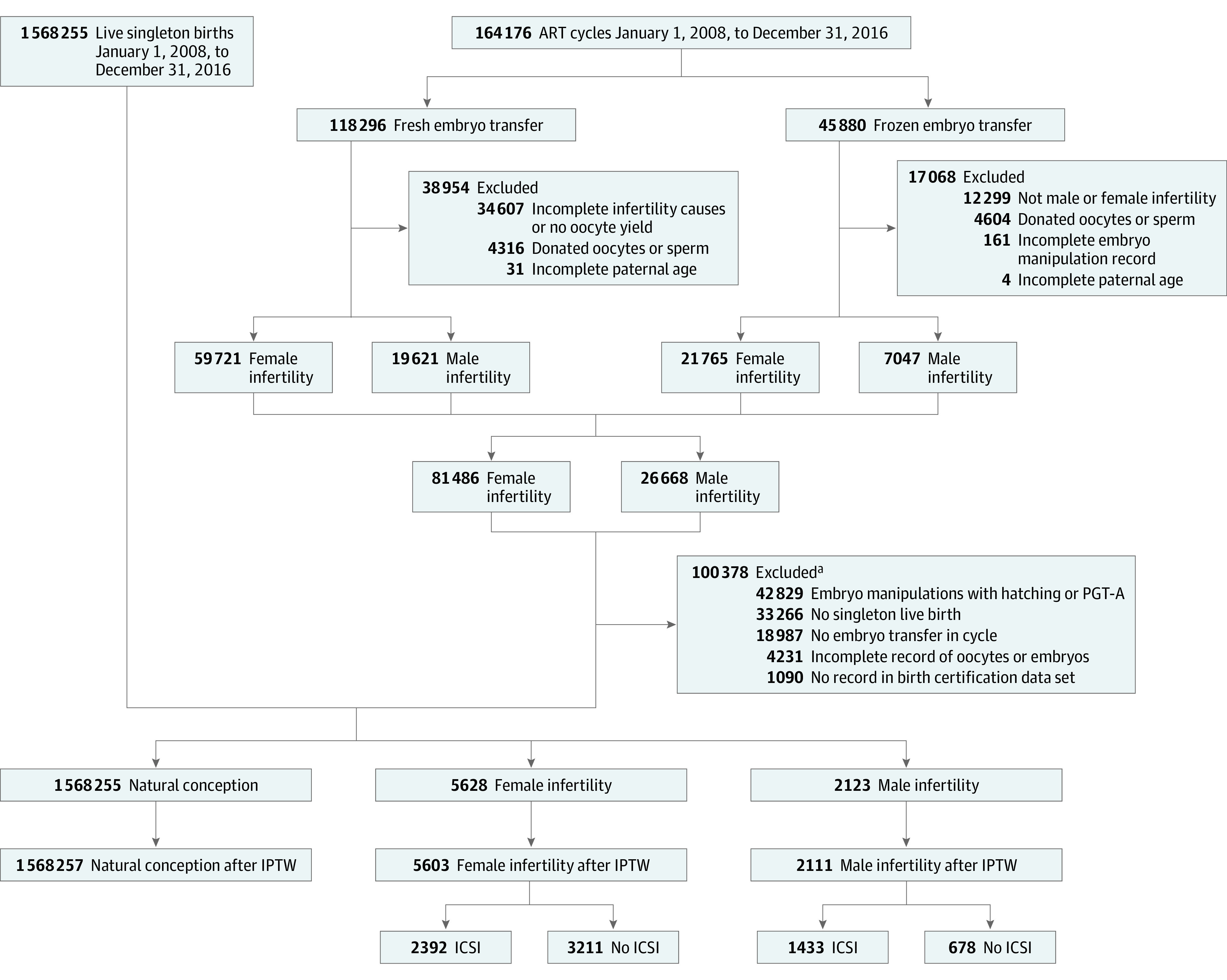
Study Flowchart ART indicates assisted reproductive technology; ICSI, intracytoplasmic sperm injection; IPTW, inverse probability of treatment weighting; PGT-A, preimplantation genetic testing for aneuploidy. ^a^See eTable 1 in Supplement 1 for additional details.

The final cohort included 1 575 971 singleton births, of whom 1 568 257 (99.5%) had natural conception, 2111 (0.1%) had ART conception associated with male infertility, and 5603 (0.4%) had ART conception associated with female infertility (Table 1). There were 756 617 girls (48.0%) and 819 389 boys (52.0%), with a mean (SD) age of 5.87 (2.60) years.

**Table 1.** Clinical Characteristics Before and After IPTW

Characteristic	Before IPTW							After IPTW						
	Patients, No. (%)							Patients, No. (%)						
	Female infertility group^a^	Male infertility group^b^	Natural conception^c^	*P* value	SMD			Female infertility group^a^	Male infertility group^b^	Natural conception^c^	*P* value	SMD		
					1 vs 2	1 vs 3	2 vs 3					1 vs 2	1 vs 3	2 vs 3
Total No.	5628	2123	1 568 255	NA	NA	NA	NA	5603	2111	1 568 257	NA	NA	NA	NA
Paternal psychiatric disorder history														
No	5500 (97.7)	2072 (97.6)	1 551 895 (99.0)	<.001	<0.001	0.097	0.105	5544 (98.9)	2088 (98.9)	1 551 799 (98.9)	.98	<0.001	0.001	0.004
Yes	128 (2.3)	51 (2.4)	16 360 (1.0)					59 (1.1)	23 (1.1)	16 458 (1.1)				
Maternal psychiatric disorder history														
No	5496 (97.6)	2072 (97.6)	1 546 894 (98.6)	<.001	<0.001	0.073	0.077	5521 (98.5)	2081 (98.6)	1 546 819 (98.6)	.79	0.003	0.009	0.005
Yes	132 (2.4)	51 (2.4)	21 361 (1.4)					82 (1.5)	30 (1.4)	21 438 (1.4)				
Risk factors during pregnancy														
Diabetes or gestational diabetes	237 (4.2)	77 (3.6)	21 663 (1.4)	<.001	0.030	0.172	0.144	86 (1.5)	32 (1.5)	21 871 (1.4)	.60	0.001	0.012	0.011
Hypertensive disorder	154 (2.7)	62 (2.9)	18 635 (1.2)	<.001	<0.001	0.112	0.122	73 (1.3)	29 (1.4)	18 759 (1.2)	.56	<0.001	0.009	0.018
Unhealthy lifestyle	3 (0.1)	0	1055 (0.1)	.48	0.033	<0.001	<0.001	4 (0.1)	0 (0.0)	1053 (0.1)	.49	0.038	0.001	<0.001
Complications during labor														
Prolonged premature rupture of membranes	189 (3.4)	50 (2.4)	26 040 (1.7)	<.001	0.060	0.109	0.050	100 (1.8)	35 (1.7)	26 151 (1.7)	.81	0.009	0.009	<0.001
Placental abruption	46 (0.8)	19 (0.9)	5099 (0.3)	<.001	<0.001	0.065	0.073	23 (0.4)	9 (0.4)	5140 (0.3)	.46	0.001	0.014	0.013
Placenta previa	253 (4.5)	56 (2.6)	11 615 (0.7)	<.001	0.101	0.237	0.148	45 (0.8)	18 (0.8)	11 867 (0.8)	.82	<0.001	0.006	0.009
Postpartum hemorrhage	80 (1.4)	27 (1.3)	5665 (0.4)	<.001	0.013	0.113	0.101	22 (0.4)	10 (0.5)	5745 (0.4)	.71	<0.001	0.005	0.015
Fetal distress	128 (2.3)	54 (2.5)	17 450 (1.1)	<.001	<0.001	0.090	0.107	66 (1.2)	26 (1.2)	17 546 (1.1)	.84	<0.001	0.006	0.009

Abbreviations: IPTW, inverse probability treatment weighting; NA, not applicable; SMD, standardized mean difference.

^a^ Represents 1 in the SMD columns.

^b^ Represents 2 in the SMD columns.

^c^ Represents 3 in the SMD columns.

Before IPTW, the natural conception group had substantially fewer risk factors for ASD, ADHD, and developmental delay than the male and female infertility groups. The risk factors included maternal or paternal psychiatric disorder history, maternal risk factors during pregnancy (eg, diabetes or gestational diabetes and hypertensive disorder), and maternal complications during labor (prolonged premature rupture of membrane, placental abruption, placenta previa, postpartum hemorrhage, and fetal distress). After IPTW, there were no significant differences in risk factors during pregnancy, complications during labor, and paternal or maternal psychiatric disorder history among the male and female infertility groups and natural conception groups. The SMD for each covariate was balanced after IPTW among the 3 groups (eTable 3 in Supplement 1; Table 1).

### Risks of Neurodevelopmental Disorders After ART With or Without ICSI

We assessed the risks of ASD, ADHD, and developmental delay in offspring in the natural conception group (n = 1 568 257) and in the groups with ICSI (n = 3825) and without ICSI (n = 3889) (Table 2). The risks of ASD (adjusted HR, 2.49; 95% CI, 1.61-3.84; *P* < .001) and developmental delay (adjusted HR, 1.92; 95% CI, 1.54-2.39; *P* < .001) were associated with ICSI use. The risk of ADHD was not significantly different between the group with ICSI and the natural conception group (adjusted HR, 1.29; 95% CI, 0.81-2.06; *P* = .28). The risks of ASD, ADHD, and developmental delay were not significantly different between the group without ICSI and the natural conception group.

**Table 2.** Incidence of ASD, ADHD, and Developmental Delay Associated With Natural Conception^a^

	No. of patients	Incidence, No. (%)	Total No. of person-years	Incidence rate per 1000 person-years	HR (95% CI)	*P* value	aHR (95% CI)	*P* value
**ASD**								
Natural conception	1 568 257	4096 (0.3)	10 049 977	0.41	1 [Reference]	NA	1 [Reference]	NA
Infertility group								
Female	5603	19 (0.3)	27 913	0.67	1.88 (1.19-2.96)	.007	1.48 (0.94-2.33)	.09
Male	2111	11 (0.5)	11 207	1.02	2.74 (1.53-4.88)	.001	2.20 (1.23-3.93)	.008
ART conception								
With ICSI	3825	21 (0.5)	18 682	1.11	3.11 (2.02-4.78)	<.001	2.49 (1.61-3.84)	<.001
Without ICSI	3889	9 (0.2)	20 142	0.47	1.26 (0.67-2.39)	.48	0.99 (0.52-1.88)	.98
**ADHD**								
Natural conception	1 568 257	10 611 (0.7)	10 036 786	1.06	1 [Reference]	NA	1 [Reference]	NA
Infertility group								
Female	5603	22 (0.4)	27 903	0.77	1.00 (0.66-1.53)	.99	1.01 (0.66-1.55)	.95
Male	2111	14 (0.7)	11 199	1.23	1.43 (0.84-2.42)	.19	1.43 (0.85-2.43)	.18
ART conception								
With ICSI	3825	18 (0.5)	18 880	0.94	1.26 (0.79-2.01)	.33	1.29 (0.81-2.06)	.28
Without ICSI	3889	18 (0.5)	20.222	0.87	1.03 (0.65-1.64)	.91	1.03 (0.64-1.64)	.92
**Developmental delay **								
Natural conception	1 568 257	22 954 (1.5)	9 984 875	2.30	1 [Reference]	NA	1 [Reference]	NA
Infertility group								
Female	5603	90 (1.6)	27 736	3.25	1.45 (1.18-1.78)	.001	1.40 (1.14-1.72)	.002
Male	2111	43 (2.0)	11 088	3.89	1.71 (1.27-2.31)	<.001	1.68 (1.25-2.27)	.001
ART conception								
With ICSI	3825	81 (2.1)	18 682	4.36	1.94 (1.56-2.41)	<.001	1.92 (1.54-2.39)	<.001
Without ICSI	3889	52 (1.3)	20 142	2.57	1.14 (0.87-1.49)	.36	1.09 (0.83-1.43)	.54

Abbreviations: ADHD, attention-deficit/hyperactivity disorder; aHR, adjusted hazard ratio; ART, assisted reproductive technology; ASD, autism spectrum disorder; HR, hazard ratio; ICSI, intracytoplasmic sperm injection.

^a^ Adjusted for maternal age, paternal age, gestation status (full-term or preterm), and offspring sex (boy or girl).

### Risks of Neurodevelopmental Disorders With or Without ICSI 

#### Female Infertility Group 

We evaluated the risks of ASD, ADHD, and developmental delay in the natural conception (n = 1 568 257), female infertility without ICSI (n = 3211), and female infertility with ICSI (n = 2392) groups (Table 2 and Table 3). In total, 4096 offspring (0.3%; 0.41 incidence per 1000 person-years) with natural conception developed ASD during the follow-up period. In the female infertility group, 19 offspring (0.3%; 0.67 incidence per 1000 person-years) developed ASD, but the risk was not significantly different compared with that in the natural conception group (adjusted HR, 1.48; 95% CI, 0.94-2.33; *P* = .09). The female infertility group was further divided into 2 subgroups based on ICSI. The incidence and risk of developing ASD were higher in the female infertility with ICSI group vs the natural conception group (12 of 2392 [0.5%]; 1.06 incidence per 1000 person-years; adjusted HR, 2.50; 95% CI, 1.41-4.42; *P* = .002). The incidence and risk were not significantly different between the female infertility without ICSI and the natural conception groups (0.2%; 0.40 incidence per 1000 person-years; adjusted HR, 0.87; 95% CI, 0.41-1.84; *P* = .71).

**Table 3.** Risks of ASD, ADHD, and Developmental Delay Associated With ICSI^a^

	No. of event/Total No. (%)	Total No. of person-year	Incidence rate per 1000 person-year	HR (95% CI)	*P* value	aHR (95% CI)	*P* value
**ASD**							
Natural conception	4096/1 568 257 (0.3)	10 049 977	0.41	1 [Reference]	NA	1 [Reference]	NA
Female							
Without ICSI	7/3211 (0.2)	16 733	0.40	1.09 (0.52-2.32)	.82	0.87 (0.41-1.84)	.71
With ICSI	12/2392 (0.5)	11 130	1.06	3.18 (1.80-5.63)	<.001	2.50 (1.41-4.42)	.002
Male							
Without ICSI	3/678 (0.4)	3455	0.76	2.10 (0.63-7.03)	.23	1.59 (0.48-5.31)	.45
With ICSI	9/1433 (0.6)	7752	1.14	3.01 (1.55-5.82)	.001	2.49 (1.28-4.81)	.007
**ADHD**							
Natural conception	10 611/1 568 257 (0.7)	10 036 786	1.06	1 [Reference]	NA	1 [Reference]	NA
Female							
Without ICSI	13/3211 (0.4)	16 767	0.80	0.95 (0.56-1.62)	.85	0.95 (0.56-1.62)	.85
With ICSI	8/2392 (0.3)	11 136	0.73	1.11 (0.56-2.21)	.76	1.14 (0.57-2.27)	.71
Male							
Without ICSI	4/678 (0.6)	3455	1.20	1.42 (0.55-3.72)	.47	1.39 (0.53-3.63)	.50
With ICSI	10/1433 (0.7)	7744	1.24	1.43 (0.76-2.69)	.27	1.46 (0.77-2.74)	.25
**Developmental delay **							
Natural conception	22 954/1 568 257 (1.5)	9 984 875	2.30	1 [Reference]	NA	1 [Reference]	NA
Female							
Without ICSI	43/3211 (1.3)	16 695	2.58	1.14 (0.85-1.54)	.39	1.09 (0.56-2.12)	.57
With ICSI	47/2392 (2.0)	11 039	4.25	1.92 (1.44-2.56)	<.001	1.90 (1.43-2.53)	<.001
Male							
Without ICSI	9/678 (1.3)	3445	2.51	1.13 (0.58-2.20)	.72	1.09 (0.56-2.21)	.80
With ICSI	35/1433 (2.4)	7683	4.52	1.97 (1.41-2.75)	<.001	1.95 (1.40-2.73)	<.001

Abbreviations: ADHD, attention-deficit/hyperactivity disorder; aHR, adjusted hazard ratio; ASD, autism spectrum disorder; HR, hazard ratio; ICSI, intracytoplasmic sperm injection.

^a^ Adjusted for maternal age, paternal age, gestation status (full-term or preterm), and offspring sex (boy or girl).

In total, 10 611 offspring (0.7%; 1.06 incidence per 1000 person-years) with natural conception developed ADHD. In the female infertility group, 22 offspring (0.4%; 0.77 incidence per 1000 person-years) developed ADHD, with no significant difference in risk compared with that in the natural conception group (adjusted HR, 1.01; 95% CI, 0.66-1.55; *P* = .95). Compared with the natural conception group, there were no significant differences in the incidence and risk of ADHD between the female infertility with ICSI group (0.3%; 0.73 incidence per 1000 person-years; adjusted HR, 1.14; 95% CI, 0.57-2.27; *P* = .71) and female infertility without ICSI group (0.4%; 0.80 incidence per 1000 person-years; adjusted HR, 0.95; 95% CI, 0.56-1.62; *P* = .85).

Overall, 22 954 offspring (1.5%; 2.30 incidence per 1000 person-years) with natural conception experienced developmental delay. In the female infertility group, 90 offspring (1.6%; 3.25 incidence per 1000 person-years) experienced developmental delay, which was significantly higher than the incidence in the natural conception group; the risk of developmental delay was also higher (adjusted HR, 1.40; 95% CI, 1.14-1.72; *P* = .002). The incidence and risk of developmental delay were significantly higher in the female infertility with ICSI group vs the natural conception group (47 of 2392 [2.0%]; 4.25 incidence per 1000 person-years; adjusted HR, 1.90; 95% CI, 1.43-2.53; *P* < .001). However, there was no significant difference in incidence or risk between the female infertility without ICSI and the natural conception groups (1.3%; 2.58 incidence per 1000 person-years; adjusted HR, 1.09; 95% CI, 0.56-2.12; *P* = .57).

#### Male Infertility Group 

We identified the implications of ICSI for the occurrence of neurodevelopmental disorders in the natural conception (n = 1 568 257), male infertility without ICSI (n = 678), and male infertility with ICSI (n = 1433) groups (Table 2 and Table 3). In total, 11 of 2111 offspring (0.5%) in the male infertility group developed ASD (1.02 incidence per 1000 person-years) vs 4096 (0.3%) in the natural conception group (0.41 incidence per 1000 person-years), and the risk of ASD was significantly higher compared with that in the natural conception group (adjusted HR, 2.20; 95% CI, 1.23-3.93; *P* = .008). The incidence and risk of ASD were higher in the male infertility with ICSI group vs the natural conception group (9 of 1433 [0.6%]; 1.14 incidence per 1000 person-years; adjusted HR, 2.49; 95% CI, 1.28-4.81; *P* = .007). However, there was no significant difference in the incidence and risk of ASD between the male infertility without ICSI group and the natural conception group (0.4%; 0.76 incidence per 1000 person-years; adjusted HR, 1.59; 95% CI, 0.48-5.31; *P* = .45).

Overall, 10 611 offspring (0.7%; 1.06 incidence per 1000 person-years) with natural conception developed ADHD. In the male infertility group, 14 offspring (0.7%; 1.23 incidence per 1000 person-years) developed ADHD, with no significant difference in the risk of ADHD vs the natural conception group (adjusted HR, 1.43; 95% CI, 0.85-2.43; *P* = .18). Furthermore, compared with the natural conception group, there were no significant differences in the incidence and risk of ADHD in the male infertility with ICSI (0.7%; 1.25 incidence per 1000 person-years; adjusted HR, 1.46; 95% CI, 0.77-2.74; *P* = .25) and male infertility without ICSI groups (0.6%; 1.20 incidence per 1000 person-years; adjusted HR, 1.39; 95% CI, 0.53-3.63; *P* = .50).

A total of 43 offspring (2.0%; 3.89 incidence per 1000 person-years); in the male infertility group had developmental delay, which was higher than the incidence in the natural conception group (22 954 [1.5%]; 2.30 incidence per 1000 person-years). The risk of developmental delay was significantly higher vs the natural conception group (adjusted HR, 1.68; 95% CI, 1.25-2.27; *P* = .001). The incidence and risk of developmental delay were significantly higher in the male with ICSI groups vs the natural conception group (35 of 1433 [2.4%]; 4.52 incidence per 1000 person-years; adjusted HR, 1.95; 95% CI, 1.40-2.73; *P* < .001). However, there was no significant difference in incidence and risk between the male without ICSI group vs the natural conception group (1.3%; 2.51 incidence per 1000 person-years; adjusted HR, 1.09; 95% CI, 0.56-2.21; *P* = .80).

## Discussion

Male infertility has been associated with offspring health through transmitted epigenetic modifications.^18,19^ The function of epigenome of sperm is affected by oxidative stress,^20^ which has been associated with increased DNA damage, lower sperm quality, and eventually male infertility.^21,22^ Oxidative stress in sperm can be an adverse factor in embryonic development, which in turn affects offspring health. These outcomes may manifest as genetic disorders, neurodevelopmental disorders, and childhood cancer.^22,23^ However, given the results of this cohort study, we could not conclude whether male infertility (vs natural conception) was associated with a higher risk of ASD and developmental delay, likely because of the use of conventional sperm assessment to detect male infertility in Taiwan. This method limited our ability to evaluate the severity of male infertility and function of sperm. Previous studies have not reported a strong correlation between paternal sperm parameters and birth defect or genetic defect in offspring.^6,24^ Additional studies are warranted to clarify whether male infertility is a risk factor for neurodevelopmental disorders in offspring.

Couples with severe male infertility experienced difficulty in achieving pregnancy via conventional IVF; however; this situation improved with the introduction of ICSI in 1992.^25^ Azoospermia, asthenozoospermia, and severe oligozoospermia (sperm count <5 million/mL) in the ejaculated semen are standardized indications for ICSI intervention.^7^ However, it remains unclear whether ICSI should be applied in other factors of male infertility, such as isolated teratozoospermia (normal sperm morphology <5%) and moderate oligoasthenoteratozoospermia (sperm concentration of 5-10 × 10^6^/mL; progressive motility <32%).^8^ According to the 2021 World Health Organization guidelines, several new techniques, such as sperm DNA fragmentation and reactive oxygen species, have been developed for detecting sperm function.^26^ Large studies are required for these new techniques to assess the ART outcome in male infertility associated with ICSI vs conventional IVF.

The implications of ICSI for the neurodevelopmental health of offspring has been described.^13^ Intracytoplasmic sperm injection bypasses the step of penetrating the zona pellucida via a micropipette to achieve oocytes fertilization. It is not possible to select the best sperm through current sperm isolation methods as they increase oxidative stress and DNA damage. Furthermore, the selected sperm might carry genetic defects and fragmented DNA, and the ICSI procedure itself (temperature, gas concentration, and pH) may increase stress to sperm and oocytes.^7,8^ Based on these findings, ICSI might be associated with increased sperm DNA damage and risks to the neurodevelopmental health of offspring.

However, ICSI does help with severe male infertility types (azoospermia and severe oligozoospermia) and female infertility with previous fertilized failure to achieve pregnancy.^7,27^ Intracytoplasmic sperm injection is believed to overcome the difficulty of sperm-oocyte interaction (ie, the defective function of penetrating the zona pellucida in poor-quality sperm and the quality of the zona pellucida is affected by poor-quality oocytes).^7,28,29^ There is no consensus of indications for ICSI use in male and female infertility. Hence, developing an efficient isolation method of sperm; providing relevant, appropriate, high-quality training for ICSI manipulation; and defining the indications of ICSI in patients with infertility are essential future strategies.

### Strengths and Limitations

The main strength of this study was the use of the national population registry, ART, and birth certification data sets in Taiwan. However, there were several limitations as well. First, semen parameter, grading of oocytes, and grading of embryos were not recorded in the ART database. Second, conventional sperm isolation methods limited the ability to ascertain the severity of male infertility. Third, there was minimal information regarding ovarian stimulation protocols and endometrial preparation methods. Fourth, we could not identify the risks of neurodevelopmental disorders in offspring with both parents experiencing infertility. Fifth, indications for ICSI in cases of male and female infertility remain unclear.

These limitations increased the potential bias to establish the association between male infertility and neurodevelopmental disorders in offspring. Additionally, it was difficult to assess whether the severity of male infertility played a role in the neurodevelopmental disorders in offspring.

## Conclusions

In this cohort study, we found that, compared with natural conception, ART with ICSI was associated with risks of ASD and developmental delay in offspring. The risks of ASD and developmental delay in offspring were significantly higher in couples with male or female infertility who used ICSI. These results suggest that ICSI is a major risk factor for neurodevelopmental disorders in offspring. However, we could not identify the implication of male infertility for the offspring neurodevelopmental disorders due to the limitation of the data sets.

Considering the fear of fertilization and pregnancy failure with ART, the popularity of ICSI is increasing worldwide. However, we reported the association of ICSI with offspring health, which may affect the established benefits of ART with ICSI. These results suggest the importance of establishing the indications of ICSI use in couples experiencing infertility.

## Notes

Supplement 1. **eTable 1.** Characteristics Presented in Female/Male Infertility With/Without ICSI Use **eTable 2.** The ART Outcome Presented in Female/Male Infertility With Fresh/Frozen Embryo Transfer and Presence/Absence ICSI Use **eTable 3.** Characteristics Presented in Female Infertility, Male Infertility, and Natural Conception Before and After IPTW

Supplement 2.
Data Sharing Statement
