# Supplementary material for: Neurodevelopmental Disorders in Offspring Conceived via In Vitro Fertilization vs Intracytoplasmic Sperm Injection
Source: JAMA Netw Open. 2022 Dec 22;5(12):e2248141. doi: 10.1001/jamanetworkopen.2022.48141 (PMC9856957; doi:10.1001/jamanetworkopen.2022.48141)
Supplement: Supplement 1. — eTable 1. Characteristics Presented in Female/Male Infertility With/Without ICSI Use eTable 2. The ART Outcome Presented in Female/Male Infertility With Fresh/Frozen Embryo Transfer and Presence/Absence ICSI Use eTable 3. Characteristics Presented in Female Infertility, Male Infertility, and Natural Conception Before and After IPTW [file jamanetwopen-e2248141-s001.pdf]

Supplemental Online Content

Lo H, Weng SF, Tsai EM. Neurodevelopmental disorders in offspring conceived via in vitro fertilization vs intracytoplasmic sperm injection. *JAMA Netw Open*. 2022;5(12):e2248141. doi:10.1001/jamanetworkopen.2022.48141

eTable 1. Characteristics Presented in Female/Male Infertility With/Without ICSI Use

eTable 2. The ART Outcome Presented in Female/Male Infertility With Fresh/Frozen Embryo Transfer and Presence/Absence ICSI Use

eTable 3. Characteristics Presented in Female Infertility, Male Infertility, and Natural Conception Before and After IPTW

This supplemental material has been provided by the authors to give readers additional information about their work.

© 2022 Lo H et al. *JAMA Network Open.*

eTable 1: Characteristics presented in female/male infertility with/without ICSI use												
	Fresh embryo transfer						Frozen embryo transfer					
	Female infertility			Male infertility			Female infertility			Male infertility		
	Patients, No. (%)		p-value	Patients, No. (%)		p-value	Patients, No. (%)		p-value	Patients, No. (%)		p-value
	No_ICSI (10,689)	ICSI (14,171)		No_ICSI (1,150)	ICSI (7,240)		No_ICSI (6,079)	ICSI (532)		No_ICSI (1,876)	ICSI (370)	
Female's age												
<30	984 (9.2)	835 (5.9)	<0.001	84 (7.3)	821 (11.3)	<0.001	549 (9.0)	56 (10.5)	0.407	224 (11.9)	45 (12.1)	0.216
30-34	3,777 (35.3)	4,022 (28.3)		433 (37.7)	3,021 (41.7)		2,270 (37.3)	182 (34.2)		749 (39.9)	163 (44.1)	
35-39	4,091 (38.3)	5,819 (41.1)		518 (45.0)	2,681 (37.0)		2,463 (40.5)	219 (41.2)		770 (41.1)	131 (35.4)	
≧40	1,837 (17.2)	3,495 (24.7)		115 (10.0)	717 (10.0)		797 (13.2)	75 (14.1)		133 (7.1)	31 (8.4)	
Male's age												
<30	331 (3.1)	278 (2.0)	<0.001	21 (1.8)	206 (2.9)	0.005	154 (2.5)	24 (4.5)	0.012	42 (2.2)	9 (2.4)	0.957
30-39	6,682 (62.5)	8,091 (57.1)		712 (61.9)	4,549 (62.8)		3,834 (63.1)	342 (64.3)		1,184 (63.1)	237 (64.1)	
40-49	3,445 (32.2)	5,252 (37.1)		383 (33.3)	2,164 (29.9)		1,966 (32.3)	161 (30.3)		573 (30.6)	108 (29.2)	
≧50	231 (2.2)	550 (3.8)		34 (3.0)	321 (4.4)		125 (2.1)	5 (0.9)		77 (4.1)	16 (4.3)	
Duration of infertility, mean (SD)/median (IQR), y	4.0 (3.1)/3.0 (3.0)	3.9(3.0)/3.0 (3.0)	<0.001	4.1 (3.0)/3.0 (3.0)	3.7 (3.0)/3.0 (3.0)	<0.001	4.0 (2.8)/3.0 (3.0)	4.0 (2.8)/3.0 (3.0)	0.814	3.5 (2.7)/3.0 (3.0)	3.5 (2.5)/3.0 (3.0)	0.144
Previous IVF cycle, mean (SD)/median (IQR), cycles	0.8 (1.5)/ 0.0 (1.0)	0.9 (1.8)/ 0.0 (1.0)	<0.001	0.9 (1.9)/ 0.0 (1.0)	0.9 (1.7)/ 0.0 (1.0)	0.701	1.9 (1.6)/1.0 (1.0)	1.9 (1.6)/1.0 (1.0)	0.912	1.9 (1.6)/1.0 (1.0)	1.8 (1.3)/1.0 (1.0)	0.458
Number of oocyte retrieved, mean (SD)/median (IQR), numbers	9.4 (6.4)/8.0 (7.0)	8.8 (6.3)/7.0 (8.0)	<0.001	9.3 (5.6)/8.0 (7.0)	11.0 (6.5)/10.0 (8.0)	<0.001						
Number of fertilized oocytes, mean (SD)/median (IQR), numbers	6.9 (4.9)/6.0 (6.0)	5.9 (4.3)/5.0 (5.0)	<0.001	6.5 (4.1)/6.0 (4.0)	6.9 (4.4)/6.0 (5.0)	0.004						
Fertilization rate (2PN zygotes/oocytes), mean (SD)/median (IQR), %	0.8 (0.2)/0.8 (0.3)	0.7 (0.2)/0.7 (0.3)	<0.001	0.7 (0.2)/0.8 (0.3)	0.7 (0.2)/0.7 (0.3)	<0.001						
Number of embryo transfer, mean (SD)/median (IQR), numbers	2.9 (1.0)/3.0 (2.0)	2.7 (1.0)/3.0 (2.0)	<0.001	3.0 (0.9)/3.0 (2.0)	2.9 (0.9)/3.0 (2.0)	0.011	2.5 (0.9)/2.0 (1.0)	2.7 (0.9)/3.0 (1.0)	<.001	2.5 (0.9)/2.0 (1.0)	2.7 (0.8)/3.0 (1.)	<0.001
Gestational condition												
No	7,366 (68.9)	10,467 (73.9)	<0.001	714 (62.1)	4,858 (67.1)	0.001	4,056 (66.7)	390 (73.3)	0.004	1,183 (63.1)	276 (74.6)	<0.001
Singleton pregnancy	2,205 (20.6)	2,699 (19.0)		280 (24.3)	1,636 (22.6)		1,481 (24.4)	97 (18.2)		501 (26.7)	62 (16.7)	
Multiple pregnancies	1,118 (10.5)	1,005 (7.1)		156 (13.6)	746 (10.3)		542 (8.9)	45 (8.5)		192 (10.2)	32 (8.7)	

© 2022 Lo H et al. *JAMA Network Open.*

eTable2: The ART outcome presented in female/male infertility with fresh/frozen embryo transfer and presence/absence ICSI use

Biochemical pregnancy				
		Pregnancy/Total, No. (%)	Odd ratio (95% CI)	P-value
Fresh embryo transfer	Female_NoICSI	4,378/10,689 (41.0)	Reference	<0.001
	Female_ICSI	5,182/14,171 (36.6)	0.831 (0.789-0.875)	
	Male_NoICSI	556/1,150 (48.3)	Reference	<0.001
	Male_ICSI	3,118/7,240 (43.1)	0.808 (0.713-0.915)	
Frozen embryo transfer	Female_NoICSI	2,773/6,079 (45.6)	Reference	<0.001
	Female_ICSI	195/532 (36.7)	0.690 (0.574-0.829)	
	Male_NoICSI	885/1,876 (47.2)	Reference	<0.001
	Male_ICSI	138/370 (37.3)	0.666 (0.530-0.838)	
Clinical pregnancy				
		Pregnancy/Total, No. (%)	Odd ratio	P-value
Fresh embryo transfer	Female_NoICSI	3,340/10,689 (31.2)	Reference	<0.001
	Female_ICSI	3,858/14,171 (27.2)	0.823 (0.779-0.870)	
	Male_NoICSI	430/1,150 (37.4)	Reference	0.006
	Male_ICSI	2,405/7,240 (33.2)	0.833 (0.732-0.948)	
Frozen embryo transfer	Female_NoICSI	2,249/6,079 (37.0)	Reference	0.001
	Female_ICSI	157/532 (29.5)	0.713 (0.588-0.865)	
	Male_NoICSI	747/1,876 (39.8)	Reference	0.001
	Male_ICSI	112/370 (30.3)	0.656 (0.516-0.834)	
Miscarriage				
		Pregnancy/Total, No. (%)	Odd ratio	P-value
Fresh embryo transfer	Female_NoICSI	964/10,689 (9.0)	Reference	0.060
	Female_ICSI	1,378/14,171 (9.7)	1.087 (0.997-1.185)	
	Male_NoICSI	100/1,150 (8.7)	Reference	0.477
	Male_ICSI	677/7,240 (9.4)	1.083 (0.869-1.349)	
Frozen embryo transfer	Female_NoICSI	714/6,079 (11.7)	Reference	0.106
	Female_ICSI	50/532 (9.4)	0.780 (0.577-1.054)	
	Male_NoICSI	194/1,876 (10.3)	Reference	0.845
	Male_ICSI	37/370 (10.0)	0.964 (0.665-1.396)	
Live birth				
		Pregnancy/Total, No. (%)	Odd ratio	P-value
Fresh embryo transfer	Female_NoICSI	3,281/10.689 (30.7)	Reference	<0.001
	Female_ICSI	3,651/14,171 (25.8)	0.784 (0.741-0.829)	
	Male_NoICSI	426/1,150 (37.0)	Reference	0.002
	Male_ICSI	2,343/7,240 (32.4)	0.813 (0.714-0.925)	
Frozen embryo transfer	Female_NoICSI	2,000/6,079 (32.9)	Reference	0.001
	Female_ICSI	139/532 (26.1)	0.721 (0.590-0.882)	
	Male_NoICSI	686/1,876 (36.6)	Reference	<0.001
	Male_ICSI	94/370 (25.4)	0.591 (0.459-0.760)	

© 2022 Lo H et al. *JAMA Network Open.*

eTable 3: Characteristics presented in female infertility, male infertility, and natural conception before and after IPTW						
	Before IPTW			After IPTW		
	Patients, No. (%)					
	Female Infertility	Male infertility	Natural Conception	Female Infertility	Male infertility	Natural Conception
	N=5,628	N=2,123	N=1,568,255	N=5,603	N=2,111	N=1,568,257
Paternal age, mean (SD)/median (IQR), y	38.2 (4.7)/38 (6)	38.4(5.2)/38(6)	33.7(5.4)/33(7)	38.1 (4.6)/38 (6)	38.3 (5.2)/38 (6)	33.7 (5.4)/33 (7)
<30	92 (1.6)	40 (1.9)	318,459 (20.3)	95 (1.7)	42 (2.0)	318,402 (20.3)
30-39	3,573 (63.5)	1,350 (63.6)	1,051,922 (67.1)	3,585 (64.0)	1,360 (64.4)	1,051,923 (67.1)
40-4	1,860 (33.1)	665 (31.3)	185,312 (11.8)	1,824 (32.5)	646 (30.6)	185,366 (11.8)
≧50	103 (1.8)	68 (3.2)	12,562 (0.8)	99 (1.8)	63 (3.0)	12,566 (0.8)
Paternal psychiatric disorder history						
No	5,500 (97.7)	2,072 (97.6)	1,551,895 (99.0)	5,544 (98.9)	2,088 (98.9)	1,551,799 (98.9)
Yes	128 (2.3)	51 (2.4)	16,360 (1.0)	59 (1.1)	23 (1.1)	16,458 (1.1)
Maternal age, mean (SD)/median (IQR), y	35.8 (3.7)/36 (5)	35.1 (3.7)/35 (5)	31.0 (4.6)/31 (6)	35.7 (3.7)/36 (5)	35.0 (3.6)/35 (4)	31.0 (4.6)/31 (6)
<30	282 (5.0)	149 (7.0)	564,920 (36.0)	295 (5.2)	155 (7.3)	564,813 (36.0)
30-34	1,720 (30.5)	756 (35.6)	655,060 (41.8)	1,745 (31.2)	760 (36.0)	655,051 (41.8)
35-39	2,654 (47.2)	989 (46.6)	301,641 (19.2)	2,621 (46.8)	981 (46.5)	301,724 (19.2)
≧40	972 (17.3)	229 (10.8)	46,634 (3.0)	942 (16.8)	215 (10.2)	46,669 (3.0)
Maternal psychiatric disorder history						
No	5,496 (97.6)	2,072 (97.6)	1,546,894 (98.6)	5,521 (98.5)	2,081 (98.6)	1,546,819 (98.6)
Yes	132 (2.4)	51 (2.4)	21,361 (1.4)	82 (1.5)	30 (1.4)	21,438 (1.4)
Gestational age						
Preterm (<37 weeks)	683 (12.1)	205 (9.7)	108,713 (6.9)	567 (10.1)	179 (8.5)	108,862 (6.9)
Full-term (≧37 weeks)	4,945 (87.9)	1,918 (90.3)	1,459,542 (93.1)	5,036 (89.9)	1,932 (91.5)	1,459,395 (93.1)
Risk factors during pregnancy						
Diabetes Mellitus/Gestational diabetes mellitus	237 (4.2)	77 (3.6)	21,663 (1.4)	86 (1.5)	32 (1.5)	21,871 (1.4)
Hypertensive disorder	154 (2.7)	62 (2.9)	18,635 (1.2)	73 (1.3)	29 (1.4)	18,759 (1.2)
Poor life style	3 (0.1)	0 (0.0)	1,055 (0.1)	4 (0.1)	0 (0.0)	1,053 (0.1)
Complications during labor						
Prolonged premature rupture of membrane	189 (3.4)	50 (2.4)	26,040 (1.7)	100 (1.8)	35 (1.7)	26,151 (1.7)
Placenta abruption	46 (0.8)	19 (0.9)	5,099 (0.3)	23 (0.4)	9 (0.4)	5,140 (0.3)
Placenta previa	253 (4.5)	56 (2.6)	11,615 (0.7)	45 (0.8)	18 (0.8)	11,867 (0.8)
Postpartum hemorrhage	80 (1.4)	27 (1.3)	5,665 (0.4)	22 (0.4)	10 (0.5)	5,745 (0.4)
Fetal distress	128 (2.3)	54 (2.5)	17,450 (1.1)	66 (1.2)	26 (1.2)	17,546 (1.1)
Characteristics of newborns						
Sex						
Boy	2,912 (51.7)	1,064 (50.1)	815,413 (52.0)	2,863 (51.1)	1,058 (50.1)	815,423 (52.0)
Girl	2,716 (48.3)	1,059 (49.9)	752,842 (48.0)	2,740 (48.9)	1,053 (48.9)	752,834 (48.0)
Bodyweight, mean (SD)/median (IQR), kg						
Preterm (<37 weeks)	2272.7 (655.9)/2400 (808)	2303.2 (623.4)/2376 (638)	2439.0 (608.0)/2530 (655)	2311.9 (589.9)/2450 (745)	2332.5 (570.7)/2414 (630)	2438.6 (608.8)/2530 (656)

© 2022 Lo H et al. *JAMA Network Open.*

	Before IPTW			After IPTW		
	Patients, No. (%)					
	Female Infertility	Male infertility	Natural Conception	Female Infertility	Male infertility	Natural Conception
	N=5,628	N=2,123	N=1,568,255	N=5,603	N=2,111	N=1,568,257
Full-term (≧37 weeks)	3107.7 (379.6)/3100 (496)	3115.2 (379.8)/3115.5 (503)	3136.4 (369.9)/3120 (478)	3110.5 (378.6)/3100 (490)	3122.9 (378.3)/3120 (501)	3136.4 (369.9)/3120 (478)
Delivery method						
Vaginal delivery	2,722 (48.4)	1,205 (56.8)	1,029,876 (65.7)	2,844 (50.8)	1,245 (59.0)	1,029,622 (65.7)
Cesarean section	2,906 (51.6)	918 (43.2)	538,379 (34.3)	2,759 (49.2)	866 (41.0)	538,635 (34.3)
1 min of Apgar score, mean (SD)median(IQR)						
Preterm (<37 weeks)	7.5 (1.8)/8 (2)	7.6 (1.6)/8 (2)	7.8 (1.5)/8 (1)	7.6 (1.6)/8 (2)	7.6 (1.5)/8 (2)	7.8 (1.5)/8 (1)
Full-term (≧37 weeks)	8.4 (0.8)/9 (1)	8.4 (0.8)/9 (1)	8.4 (0.7)/8 (1)	8.5 (0.8)/9 (1)	8.5 (0.8)/9 (1)	8.4 (0.7)/8 (1)
5 mins of Apgar score, mean (SD)/median (IQR)						
Preterm (<37 weeks)	8.7 (1.4)/9 (1)	8.8 (1.2)/9 (0)	8.9 (1.2)/9 (1)	8.8 (1.2)/9 (1)	8.8 (1.1)/9 (0)	8.9 (1.2)/9 (1)
Full-term (≧37 weeks)	9.3 (0.6)/9 (1)	9.3 (0.6)/9 (1)	9.3 (0.6)/9 (1)	9.3 (0.6)/9 (1)	9.3 (0.5)/9 (1)	9.3 (0.6)/9 (1)

© 2022 Lo H et al. *JAMA Network Open.*
