# Supplementary material for: Neurodevelopmental Disorders in Offspring Conceived via In Vitro Fertilization vs Intracytoplasmic Sperm Injection
Source: JAMA Netw Open. 2022 Dec 22;5(12):e2248141. doi: 10.1001/jamanetworkopen.2022.48141 (PMC9856957; doi:10.1001/jamanetworkopen.2022.48141)
Supplement: Supplement 2. — Data Sharing Statement [file jamanetwopen-e2248141-s002.pdf]

Data Sharing Statement

Lo. Neurodevelopmental Disorders in Offspring Conceived via In Vitro Fertilization vs Intracytoplasmic Sperm Injection. *JAMA Netw Open*. Published December 22, 2022. doi:10.1001/jamanetworkopen.2022.48141

Data
Data available: No
